# *Eimeria maxima*-induced transcriptional changes in the cecal mucosa of broiler chickens

**DOI:** 10.1186/s13071-019-3534-4

**Published:** 2019-06-04

**Authors:** Charles Li, Xianghe Yan, Hyun S. Lillehoj, Sungtaek Oh, Liheng Liu, Zhifeng Sun, Changqin Gu, Youngsub Lee, Zhezi Xianyu, Hongyan Zhao

**Affiliations:** 1 Animal Biosciences and Biotechnology Laboratory, Beltsville Agricultural Research Center, Agricultural Research Service, US Department of Agriculture, Beltsville, MD 20705 USA; 2 Environmental Microbial and Food Safety Laboratory, Beltsville Agricultural Research Center, Agricultural Research Service-US Department of Agriculture, Beltsville, MD 20705 USA; 3 College of Animal Science and Technology, Jiangxi Agricultural University, Nanchang, 330045 Jiangxi People’s Republic of China; 4 College of Veterinary Medicine, Huazhong Agricultural University, Wuhan, Hubei 430070 People’s Republic of China; 5 College of Veterinary Medicine, Yangzhou University, Yangzhou, 225009 Jiangsu People’s Republic of China

**Keywords:** *Eimeria maxima*, Host, Ceca, RNA-sequencing, Chicken, Transcriptome

## Abstract

**Background:**

Apicomplexan protozoans of the genus *Eimeria* cause coccidiosis, one of the most economically relevant parasitic diseases in chickens. The lack of a complete understanding of molecular mechanisms in the host-parasite interaction limits the development of effective control measures. In the present study, RNA sequencing (RNA-Seq) was applied to investigate the host mRNA profiles of the cecal mucosa collected at day 5 post-infection with *Eimeria maxima* (EM).

**Results:**

Total RNA from cecal samples of the uninfected naïve control and the EM groups was used to make libraries, generating 354,924,372 and 356,229,250 usable reads, respectively, which were assembled into a total of 386,088 high-quality unigenes (transcripts) in Trinity software. RNA-Seq analysis of cecal samples in the two groups revealed 332 upregulated and 363 downregulated genes with significant differences (*P* ≤ 0.05), including several significant immune-related gene families, such as the major histocompatibility complex (MHC) class I alpha chain, granzyme A and immunoglobulin subtype genes among upregulated differentially expressed genes. In addition, a total of 60 clusters of differentiation (CD) molecular genes and 570 novel genes were found. The completeness of the assembled transcriptome was further assessed using the Kyoto Encyclopedia of Genes and Genomes (KEGG) database, Gene ontology (GO), eggNOG and CAZy for gene annotation. The broad gene categories represented by the highly differentiated host genes suggested enrichment in immune responses, and downregulation in the metabolic pathway, MARK signaling pathway, vascular smooth muscle contraction, and proteins processing in endoplasmic reticulum after EM infection.

**Conclusions:**

*Eimeria maxima* induced statistically significant differences in the cecal mucosal gene expression of infected chickens. These findings provide new insights into the host-parasite interaction and enhance our understanding of the molecular mechanism of avian coccidiosis.

**Electronic supplementary material:**

The online version of this article (10.1186/s13071-019-3534-4) contains supplementary material, which is available to authorized users.

## Background

Coccidiosis, caused by the apicomplexan protozoan parasites of the genus *Eimeria*, is one of the most economically relevant enteric infectious diseases affecting commercial poultry, and significantly affects animal welfare and poultry production worldwide [1]. To date, nine *Eimeria* species (*E. acervulina*, *E. brunetti*, *E. maxima*, *E. necatrix*, *E. praecox*, *E. mitis*, *E. tenella*, *E. mivati* and *E. hagani*) have been identified in chickens [2, 3]. They infect and multiply within the mucosal epithelial layers in different parts of the gut *via* an oral route. Significant poultry production losses can occur from gut damage, including inflammation, hemorrhage and diarrhea, as well as high morbidity and mortality [4]. Coccidiosis is a predisposing factor for another important enteric infectious disease, necrotic enteritis, mainly caused by *Clostridium perfringens* [5, 6]. Currently, coccidiosis is controlled primarily by prophylactic coccidiostats, anticoccidial drugs that are administered in feed [7, 8]. However, the extensive use of anticoccidial drugs has led to the global emergence of antibiotic-resistant pathogens at a rate that has outpaced the development of new drugs [9, 10].

With the anticipated decrease and eventual withdrawal of anticoccidial drugs in agricultural animal production as a result of increased worldwide regulatory restrictions, the development of alternatives to antibiotics to boost host defense is a high priority [11, 12]. The immune response plays a crucial role in protecting hosts against infectious agents. A protective immune response can be achieved through genetic selection or immune modulation of the host animal. A comprehensive understanding of host immune system-parasite interaction in the gut is crucial for the design of new approaches against coccidiosis control. One study has indicated that both antibody and cell-mediated immune responses are activated after coccidiosis, although cell-mediated immunity plays a major role in disease resistance against coccidiosis [13]. Unlike those for many protozoan parasites, the primary target tissue for coccidia is the intestinal epithelium. Local gene expression changes in small intestine immune cells associated with *Eimeria maxima* (EM) and *Eimeria acervulina* infection using cDNA microarray have been profiled [14, 15]. However, no information has been reported on whether there are any host responses to EM infection in ceca. In the present study, we have focused on the ceca due to their unique physiological and immunological features. Ceca are the organs to harbor the highest microbial cell densities (up to 10^11^ cells/g), have the longest residence time (12–20 h) of digesta in the gastrointestinal tract, and are important sites for active metabolic activity including recycling of urea, water regulation and carbohydrate fermentations, contributing to intestinal health and nutrition [16–18]. In addition, the cecal tonsil acts as an important immune organ due to the presence of large masses of diffuse and nodular lymphatic tissues in the lamina propria and submucosa [1]. During infection, EM may migrate to the cecal sites, interact with other important pathogens, such as *Salmonella typhimurium* and *Clostridium perfringens*, and enhance their infections [19, 20]. According to Shiotani et al. [21], EM sporocysts released in the small intestine, especially in the jejunum, can arrive at the ceca 6 h post-infection, and about 26% of the total inoculum may migrate to the ceca 12 h post-oral-infection. Coccidiosis and necrotic enteritis have caused $2 and $6 billion economic losses annually to the worldwide poultry industry, respectively [5, 21].

In the present study, we used next-generation sequencing technology (RNA-Seq) to advance an understanding of the host-parasite interactions in the cecal mucosa and to further elucidate the fundamental immunological processes in the cecal tissues and their underlying molecular mechanisms in avian coccidiosis. RNA-Seq has become a widely used approach to study quantitative and qualitative aspects of transcriptome data for eukaryotic and prokaryotic RNA-Seq (meta-transcriptomics) experiments [22]. We performed RNA-seq to identify the profiles of mRNA which were differentially expressed in the cecal mucosa of uninfected and EM-infected chickens.

## Methods

### Animals

One-day-old Ross 708 broiler chickens (Longenecker’s Hatchery, Elizabethtown, PA, USA) were housed in Petersime starter brooder units and were provided with feed and water *ad libitum*. All birds were maintained in a temperature-controlled environment based on a standard protocol.

### Infection of chickens with *E. maxima* and RNA extraction

Chickens (15 birds per group) were randomly assigned to uninfected naïve control (N) and *E. maxima* infection (EM) groups. Briefly, chickens were infected with EM strain 41A (5000 oocysts/bird) by oral gavage on day 14 post-hatch. Uninfected control birds were mock-inoculated with an equal volume of PBS by oral gavage. The birds were fed a starter diet containing 16% crude protein and 61% carbohydrate between days 1 and 18 post-hatch, and a standard grower diet (USDA-Feed Mill, Beltsville, MD, USA) containing 24% crude protein and 54% carbohydrate between days 18 and 19. Body weights were measured on days 14 and 19 post-hatch. The chickens were closely monitored for any clinical signs. On day 19 (day 5 post-infection), all birds were euthanized, and their cecal mucosa and contents were scraped and immediately placed into liquid RNALater according to the manufacturer’s instructions (Sigma-Aldrich, St Louis, MO, USA). The RNA was extracted with an RNeasy PowerMicrobiome Kit (Qiagen Inc, Gaithersburg, MD, USA). Fresh RNA extracted from six randomly selected cecal samples were pooled, resulting in three pools per group, and sent to Novogene Inc. (Chula Vista, CA, USA) for RNA-seq.

### Library preparation, sequencing, and analysis

The RNA-sequencing service was performed by Novogene Inc, as similarly described elsewhere [23]. Briefly, before library construction, all samples were tested for: (i) RNA degradation and potential contamination by using agarose gel electrophoresis; (ii) RNA purity (OD260/OD280) by using NanoDrop spectrophotometry; (iii) RNA concentration by using Qubit fluorimetry; and (iv) RNA integrity by using an Agilent 2100 instrument. After the above QC procedures, rRNA was removed with a Ribo_Zero rRNA removal kit (Illumina Inc, San Diego, CA, USA). The enriched mRNA samples were fragmented randomly in fragmentation buffer, and cDNA synthesis was then performed by using random hexamers and M-MuLV reverse transcriptase (RNase H). After first-strand synthesis, a custom second-strand synthesis buffer (Illumina) was added, with dNTPs, RNase H and *Escherichia coli* DNA polymerase I to generate the second strand by nick-translation. AMPure XP beads were used to purify the cDNA. The final cDNA library was then ready after a round of purification, terminal repair, A-tailing, ligation of sequencing adapters, size selection and PCR enrichment. The library concentration was quantified by using a Qubit 2.0 fluorometer (Life Technologies, Carlsbad, CA, USA), diluted to 2 ng/µl before checking the insert size on an Agilent 2100 instrument, and then quantified to greater accuracy through quantitative PCR (library activity > 2 nM). Libraries were fed into HiSeq 2500 machines according to the established activity and expected data volume.

All the following analyses were based on the clean data, which were of high quality. The reference genome and gene model annotation files were downloaded from the genome website ftp://ftp.ensembl.org/pub/release-95/fasta/gallus_gallus/dna/. An index of the reference genome was built using Bowtie v.2.0.6 [24], and paired-end reads were aligned to the chicken genome (Galgal GRCg6a) with TopHat v.2.0.9 [25]. Mapped reads belonging to each sample were assembled with Cufflinks v.2.1.1 in a reference-based approach [26]. Transcripts from all samples were then merged with the Cuffmerge tool to construct a consensus set of transcripts across the samples.

### Whole-transcriptome sequencing data processing and differential gene expression analysis

The rRNA sequences of each sample were removed by mapping to an rRNA database, tRNA database and SILVA database. The whole-transcriptome sequencing data were processed and processing and differentially expressed genes were analyzed as described elsewhere [27].

### Gene ontology and KEGG pathway enrichment analysis of DEGs

Gene ontology (GO, http://www.geneontology.org/) and GO enrichment analysis were implemented using the software GOseq, topGO and *hmmscan* release 2.12 (*P* < 0.05) [28]. GO terms have been widely used to describe cellular components, molecular function and biological processes of genes. KEGG functional annotation enrichment analysis was carried out with the online software KOBAS v.2.0 (http://kobas.cbi.pku.edu.cn/home.do), and GO terms and KEGG pathways with *P*-values < 0.05 were considered significantly enriched. In this study, scatter diagram plots were used to visualize the KEGG enrichment analysis results, and the top 20 most significantly enriched pathways were chosen in the KEGG scatter plot unless the enriched pathway count was less than 20, in which case all pathways were included in the plot.

### Novel transcript identification and analysis

Novel transcript discovery was performed for each replicate by using Cufflinks v.2.1.1 (default parameter) in a reference-based approach [26]. We used rMATS v.3.0.8 (default parameter) computational pipeline to identify splicing changes [29].

## Results and discussion

### Study design

**Fig. 1:**
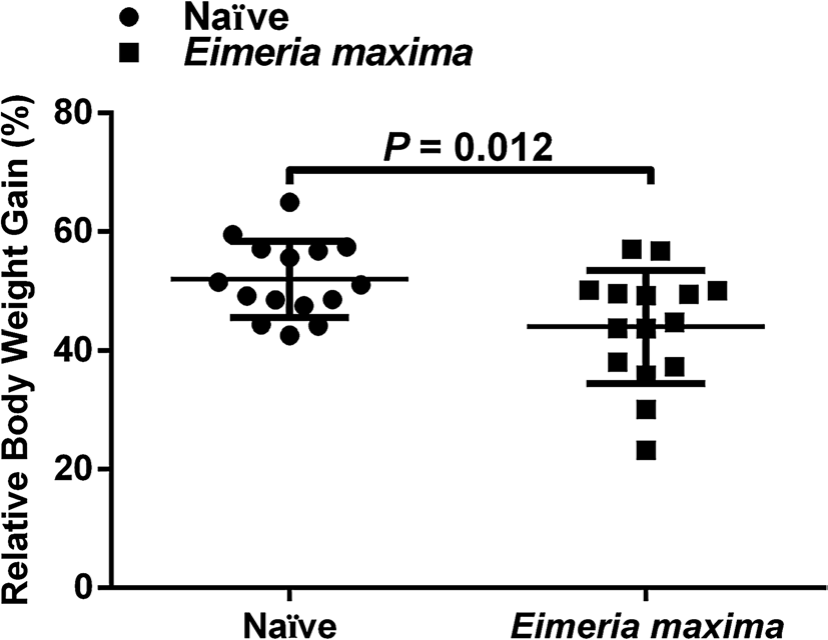
The relative body weight gain percentage (RBWG%) between uninfected naïve control (*n* = 15) and *Eimeria maxima*-infected groups (*n* = 15). The chickens were weighed prior to *Eimeria maxima* infection (EM) and at day 5 post-infection (EM5). The RBWG% was calculated by the following equation: (BW_EM5_ − BW_EM0_)/BW_EM0_ × 100%. The RBWG% is expressed as the mean (middle line) with standard errors (top and lower lines). Each data point is a single individual and the horizontal line is the mean for the respective groups of samples. The difference between the two groups is statistically significant (t-test, *t*_(28)_ = 2.702, *P* = 0.012)

Although coccidiosis, caused by *Eimeria* spp., is an important parasitic infection of chickens that is responsible for significant economic losses in the poultry industry, in-depth knowledge of the host-parasite interaction at the transcriptional level is limited. Therefore, we conducted a comprehensive transcriptomic analysis after generating RNA-Seq datasets from a total of six pooled cecal samples from EM and N chicken groups. When chickens were infected with *E. maxima*, chicken growth performance was significantly hampered as demonstrated by a much slower relative body weight gain (%) compared to those birds in the uninfected naïve control group (Fig. 1). At day 5 post-infection, infected birds showed some clinical signs, such as depression and ruffled feathers, as similarly described elsewhere [30], but no clinical signs were observed in the uninfected control group.

### Mapping and analysis of Illumina reads

Previous studies from our laboratory have provided a strong basis for biological and technical averaging and variance reduction in pooled RNA samples (data not shown). In our study, RNA samples isolated from the N and EM chickens were sequenced using the Illumina platform to generate more than 711 million high-quality clean reads representing 106.66 Gbp with an error rate of sequenced bases in all samples less than 0.02% (Additional file 1: Table S1). The Cufflinks assembler and TopHat2 were used for genome-guided assembly and mapping of all clean reads to the chicken host genome. The overview of the number of genes with differential gene expression levels relative to host *Gallus gallus* is displayed in Additional file 1: Table S2. The similar levels of relative proportions of genes with different expression levels in *Gallus gallus* were found across all fragments per kilobase of transcript per million mapped reads (FPKM) interval for each sample. In this study, novel gene prediction based on the mapping information from all samples was combined and used as input into the regular Cufflinks assembler. The assembled trans-fragments from each assembly were then compared to the reference transcripts to determine novel genomic information, which led to the discovery of novel genes and novel exons, and the optimization of the start and end information of known transcripts. The output is presented as GTF files (more information about the GTF format is available at http://mblab.wustl.edu/GTF22.html).

### Identification of differentially expressed genes (DEGs) of EM *versus* N chickens

**Fig. 2:**
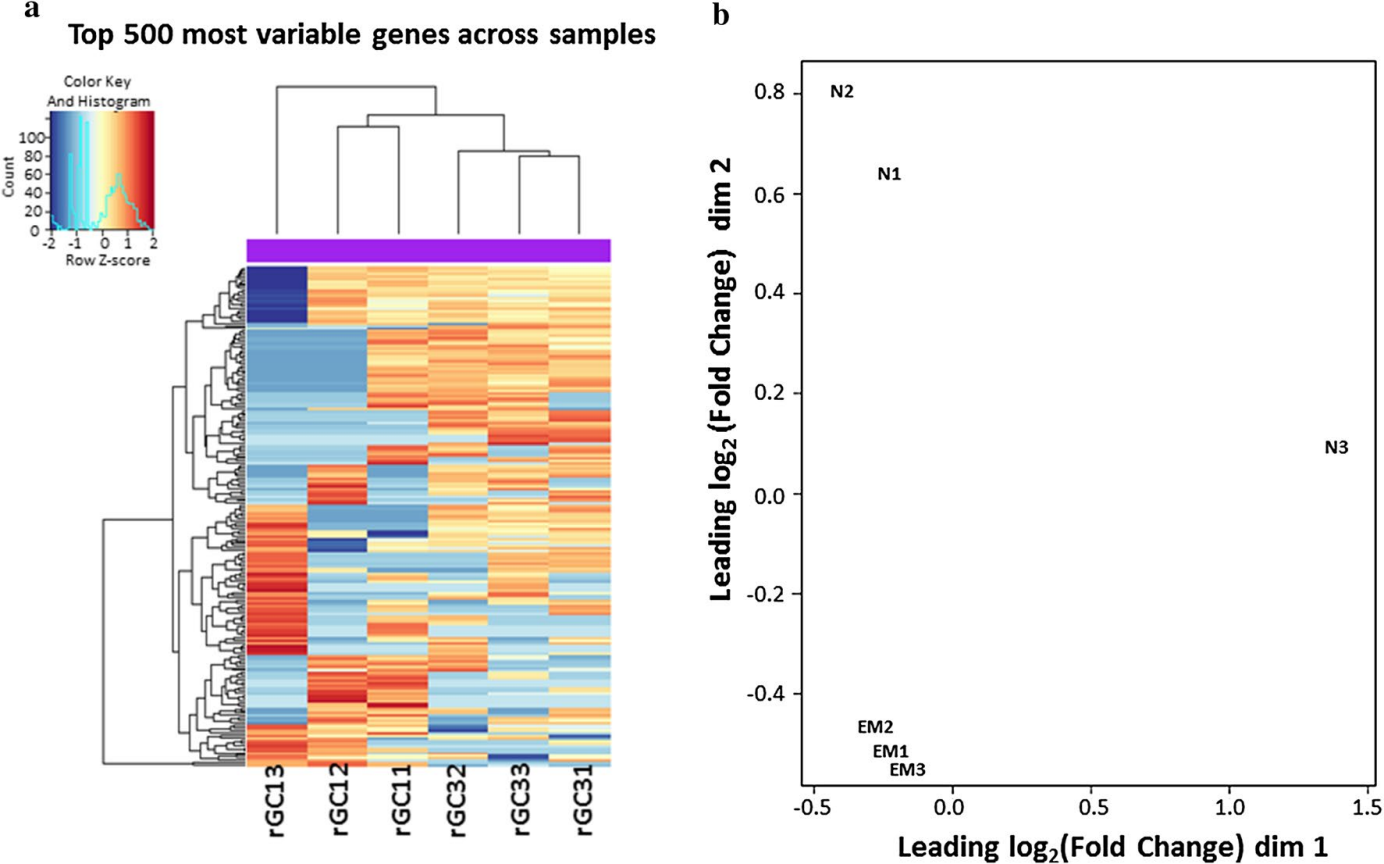
Summary of RNA-seq data in the chicken ceca between naïve (N: rGC11, rGC12 and rGC13) and *Eimeria maxima*-infected groups (EM: rGC31, rGC32 and rGC33). **a** Heat map showing normalized expression of the 500 most variable genes across all samples. Hierarchical clustering is performed using these genes and reveals that samples cluster based on a gene quantification (log2 of expression ratios). **b** The multidimensional scaling (MDS) plot of the dataset. By using a count-specific distance measure, edgeR’s plotMDS produces a MDS plot showing the relationship between all pairs of samples

To better understand coccidiosis, the read counts from gene expression level analysis were used as input data for differential gene expression analysis. The differential gene expression analysis comprised the following three steps: (i) read count normalization; (ii) negative binomial distribution model dependent *P*-value estimation; and (iii) value estimation of the false discovery rate (FDR) based on multiple hypothesis testing. DEGseq software (v.1.10.1) was used to identify the DEGs between the two different conditions. Fragments per kilobase of transcript per million base pairs sequenced were used to estimate the level of gene expression. A heat map of the top 500 most variable genes across samples is shown in Fig 2a. Figure 2b graphically illustrates the sample distributions for each group by using a multidimensional scaling plot, which was inspected using the function plotMDSown. Generally, samples in EM group were more clustered, while samples from naïve control groups were distributed widely, suggesting that the mucosal layers were more easily collected by scraping after EM infections.

**Fig. 3:**
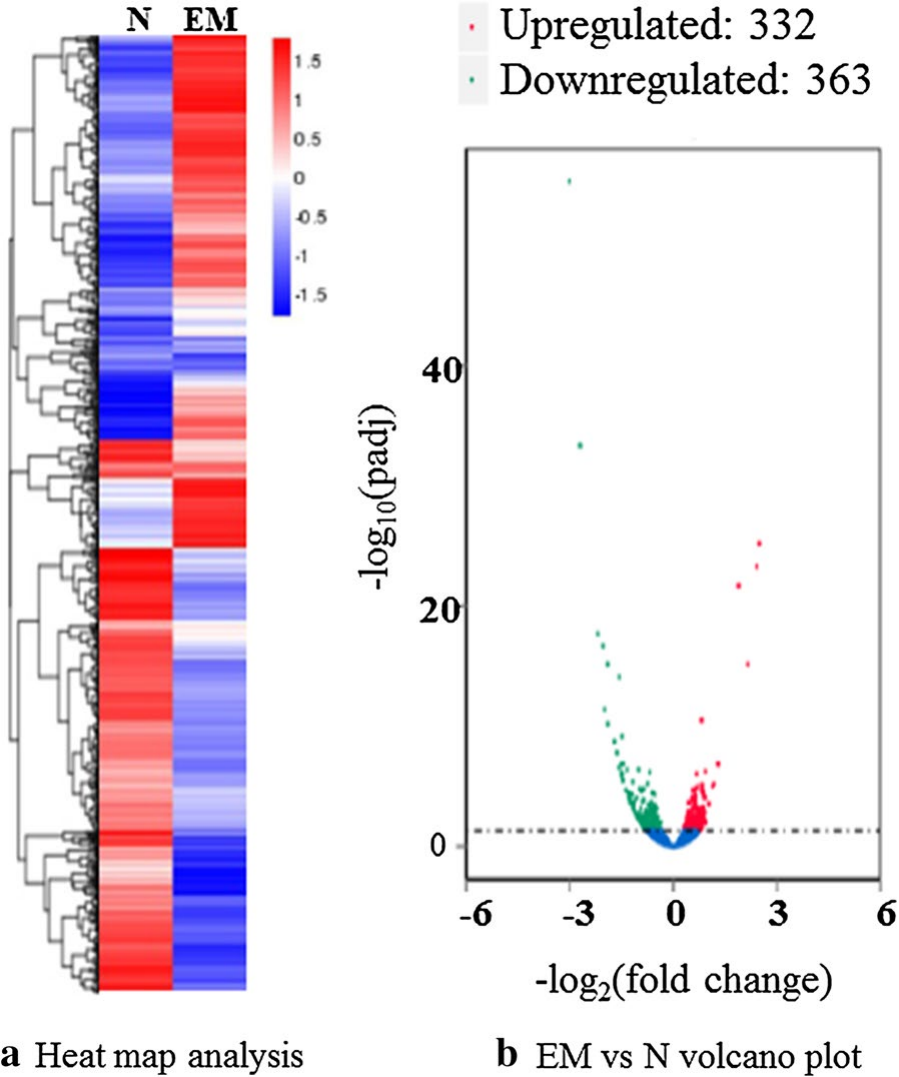
Cluster analysis of differentially expressed genes between naïve (N) *versus E. maxima* (EM) infection. **a** Heat map analysis between two groups based on the overall results of FPKM cluster analysis performed using the log10 (FPKM + 1) data. Red denotes genes with high expression levels, and blue denotes genes with low expression levels. The color range from red to blue represents the log10(FPKM + 1) value from large to small. **b** Volcano plot. The x-axis shows the fold change in gene expression between different samples, and the y-axis shows the statistically significant differences. Significantly upregulated and downregulated genes are highlighted in red and green, respectively. Genes that did not express differently between the treatment group and the control group are in blue

The heat map figure across all the genes indicates that there were significant differences in DEGs between two groups (Fig. 3a). Volcano plots indicated that RNA-Seq analysis of cecal samples in two groups revealed 332 upregulated and 363 downregulated genes (|fold change| > 1.2 to 1.27, adjusted *P* ≤ 0.05) (Fig 3b), among which 124 upregulated genes and 305 downregulated genes were identified in these comparisons (|fold change| ≥ 1.5, adjusted *P* ≤ 0.05), indicating that the numbers of downregulated DEGs were relatively increased at day 5 post-EM infection in chicken ceca. The upregulated genes with higher than 1.5-fold increase in chicken cecal epithelial mucosa are listed in Additional file 1: Table S3. These genes represent the main components of innate and adaptive immunity related to immune responses, such as major histocompatibility complex (MHC) class I alpha chain (2.27-fold to 5.60-fold increase), granzyme A (2.04-fold increase), suppressor of cytokine signaling 3 (1.88-fold increase), PANTR C-C motif chemokine 3 (1.74-fold increase), V-set and immunoglobulin domain-containing protein 4 (1.71-fold increase), innate immunity activator protein (1.68-fold increase), lymphocyte antigen 96 (1.67-fold increase), MHC Class II antigen beta chain (1.66-fold increase), programmed cell death 1 ligand (1.66-fold increase), tumor necrosis factor ligand superfamily member 10 (1.50-fold to 1.65-fold increase) and tumor necrosis factor receptor superfamily member 10B (1.61-fold increase). Two novel genes were also identified, both upregulated (1.69-fold increase for Novel00358 and 1.57-fold increase for Novel00363).

Additional file 1: Table S4 lists the significantly downregulated genes with more than 1.5-fold decrease in comparisons at day 5 post-EM infection in chicken cecal mucosa. Among the top 10 downregulated transcripts with defined functions, 4 encode proteins of rRNA processing family (RRP1, 7, 12, and 36; 4.09-fold to 8.04-fold decrease), while other 4 are involved in metabolism: fatty acid-binding protein (3.73-fold decrease), calcium-binding proteins (3.25-fold decrease), apolipoprotein B-100 (3.10-fold decrease), potassium channel voltage-dependent beta subunit (2.96-fold decrease). Chicken Histone H2A gene was also downregulated (2.86-fold decrease) and could be assumed to play an important role in transcription regulation, DNA repair, DNA replication and chromosomal stability as in mammals [31]. Interestingly, chicken NOD-like receptor (NLRC3) transcript was highly downregulated (3.98-fold decrease). Murine homolog NLRC3 is indicated to be involved in host immunity as a negative regulator of innate immunity [32]. It may be reasonable to expect the initiation of innate immunity by downregulating its negative regulator NLR3 since the innate immunity is usually the first line of defense against pathogens including *E. maxima*. These observations are in agreement with another study reporting reduced metabolism in chicken cecal epithelia in response to *Eimeria tenella* infection [33].

### GO and KEGG enrichment analysis

**Fig. 4:**
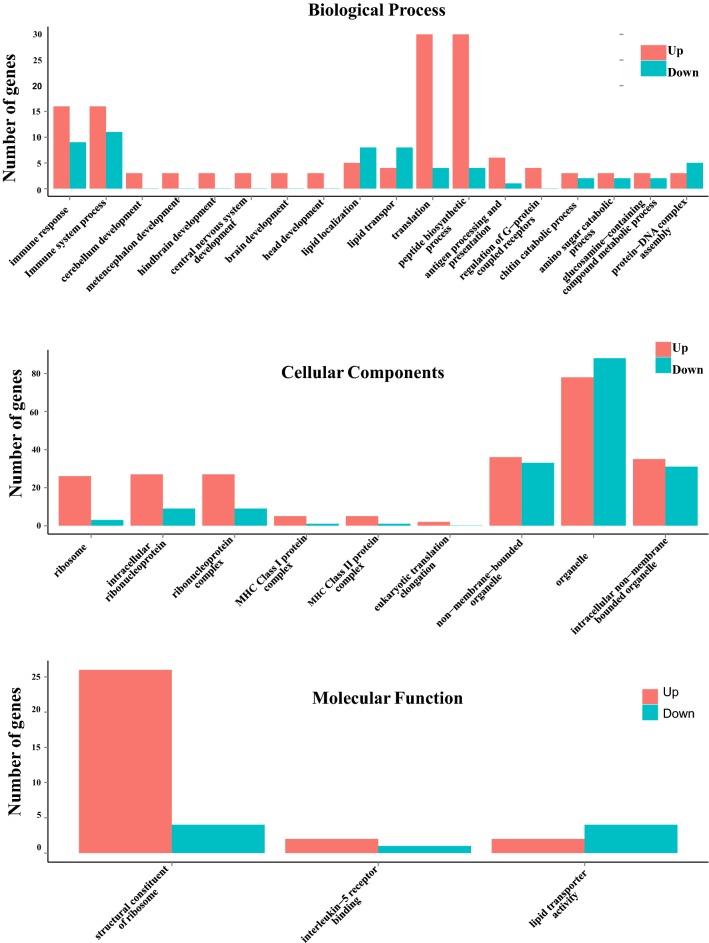
The most enriched gene ontology (GO) terms in upregulated and downregulated genes in chicken ceca for naïve (N) *vs E. maxima*-infected (EM) groups. The x-axis indicates GO terms enriched and the y-axis represents the number of differentially expressed genes. The GO terms: biological process, cellular components and molecular function are compared based on numbers of upregulated and downregulated genes between naïve (N) *vs E. maxima*-infected (EM) groups

In this study, GO term enrichment analysis and KEGG pathway analysis were applied to identify pathways in which DEGs were significantly enriched. Figure 4 shows the DEGs classified into three main categories: biological process, cellular components and molecular function. An analysis of the DEGs from the GO database comparison revealed that 18 terms of biological process category (Fig. 4) were classified: upregulated (15 terms) and downregulated (3 terms). The upregulated DEGs were significantly enriched in terms of immune responses, immune system process and translation peptide biosynthetic process, whereas the downregulated DEGs were mainly enriched in the terms of lipid localization and transport, and protein-DNA complex assembly.

Of total 9 terms of the cellular components category, 6 terms were classified by more upregulated DEG, 2 were close to even, and only one term was classified by more downregulated DEG (organelle). These 6 upregulated terms included ribosome, intracellular ribonucleoprotein, ribonucleoprotein complex, eukaryotic translation elongation, MHC class I and class II proteins which play a pivotal role in the adaptive branch of the immune system. Both classes of proteins share the task of presenting peptides on the cell surface to recognition by T cells.

Interestingly, analysis of the molecular function category showed that only one of 3 terms was classified by more downregulated DEGs (lipid transporter activity) after EM infection. These data suggest that complex gene regulatory mechanisms underlie host-parasite interaction in coccidiosis.

### Expression of DEGs in key KEGG pathways

**Fig. 5:**
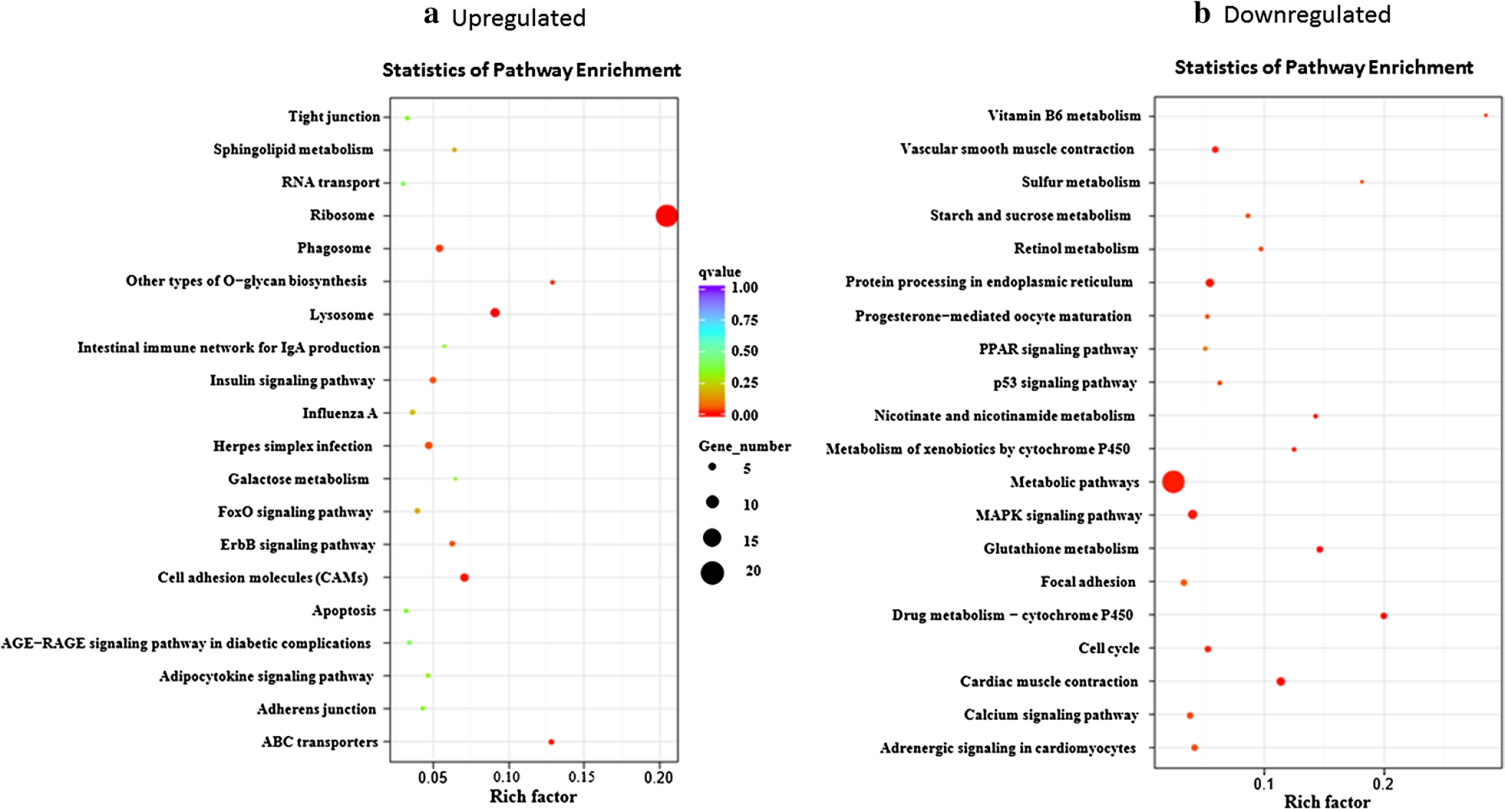
KEGG enrichment pathways analysis among the identified differentially expressed genes for naïve (N) *versus Eimeria maxima* (EM) infected groups. **a** Upregulated genes. **b** Downregulated genes. The y-axis shows the name of the pathway and the x-axis shows the Rich factor. Dot size represents the number of different genes and the color indicates the q-value. The Rich factor is the proportion of the number of differentially expressed genes and the number of all annotated genes in a given pathway. The greater the Rich factor, the higher the degree of enrichment. The q-value is the adjusted *P*-value after multiple hypothesis testing, and its range is [0, 1]. The closer the q-value is to zero, the more significant the enrichment. The top 20 most significant enriched pathways were chosen in the KEGG scatter plot, unless the enriched pathway count was less than 20, in which case all pathways were included in the plot

The KEGG pathway analysis of the differentially expressed mRNAs between EM *vs* N groups was used to perform further functional classification and pathway assignment of the upregulated and downregulated DEGs (Fig. 5a, b). The top pathways enriched in the upregulated DEGs were ribosome, lysosome, phagosome, cell adhesion molecules, insulin signaling pathway and Herpes simplex infection pathways (Fig. 5a). In contrast, the pathways enriched in the downregulated DEGs were the metabolic pathway, MARK signaling pathway, vascular smooth muscle contraction, and proteins processing in the endoplasmic reticulum (Fig. 5b). A previous report showed that the most frequent and significantly enriched biological pathways are related to metabolic processes, cell proliferation and the primary innate immune response in identifying candidate genes and genomic regions associated with traits in genotyping of blood samples using Affymetrix Axiom HD genotyping array in response to *Eimeria maxima* infection in broilers [34].

### Identification of a novel locus significantly related to host immune response

By applying a deep sequencing approach to the entire chicken transcriptome, we identified 570 previously unknown transcribed loci in the chicken genome, with unknown specific functions for most novel gene-encoded proteins. This result confirms the power of the RNA-Seq approach to reveal potential novel functional elements related to the immune response. A total of 60 molecular genes for clusters of differentiation (CD) were also found.

### Differential expression of immune response genes and metabolic dysregulation

In our earlier studies [35], pathway gene analysis from intestinal intraepithelial lymphocytes following *E. maxima* infection in chickens indicated that many of the modulated genes were related to apoptosis, JAK/STAT, MAPK, interleukin and TLR signaling pathways, and involving innate and adaptive immune responses. These pathogens elicit local inflammatory responses including production of pro-inflammatory cytokine, such as lipopolysaccharide-induced TNF factor (LITAF), IL-1β and IL-6 [36], and several β-defensins, which are known to be upregulated during inflammation [37]. For this reason, we assessed the pro-inflammatory cytokines in the N and EM groups in this study. The data are shown in Additional file 1: Table S5. Compared with pro-inflammatory cytokine expression levels between the control and EM chickens in intestinal mucosal layer, IL-6 showed no gene expression change, whereas many genes encoding components within the intestinal immune network were moderately elevated in EM group in all pooled chicken samples in response to E. maxima infection, including VEGFC (1.08-fold increase), LITAF (1.21-fold increase), TRADD (1.27-fold increase) and CD28 (1.24-fold increase). The moderate increases in the expression of these genes in the cecal epithelial layer in this study may not be as high as the transcriptional levels obtained with an Affymetrix chicken microarray in the ceca in response to infection with another species, *Eimeria tenella* [33], possibly because ceca are *E. tenella* species-specific tissues, but not the favorite specific tissues for *E. maxima*.

Although we did not analyze the whole-body tissue response in this study, our present analysis focusing on the area of the ceca demonstrates a comprehensive regulation of the immune response to *E. maxima* revealed through our approach. We intend to further investigate the detailed host-parasite interaction on the basis of the novel gene findings from our present results.

## Conclusions

We used RNA-Seq technology to obtain an overview of the dynamic expression changes in the host transcriptome during *E. maxima* infection. Through pairwise comparison analyses of DEGs between EM and N tissue samples, we identified many transcripts likely to control genomic and immune heterogeneity in coccidiosis, and many novel transcripts that may be involved in the specificity, breadth, and intensity of the immune response to coccidiosis. Our findings confirm and extend our previous results regarding the complexity of host immune responses and mechanisms of the adaptive immune response associated with protecting against intracellular protozoan infections of destructive consequences. Overall, the results of this study should facilitate further dissection of the molecular mechanisms underlying coccidiosis and provide an enhanced understanding of host-parasite interaction in coccidiosis.

## Additional file

**Additional file 1: Table S1.** Data quality control summary. **Table S2**. The number of genes with different expression levels in *Gallus gallus.* **Table S3**. List of significantly upregulated genes in chicken cecal mucosa in response to *Eimeria maxima* infection. **Table S4**. List of significantly downregulated genes in chicken cecal mucosa in response to *Eimeria maxima* infection. **Table S5**. Gene expression changes for some pro-inflammatory molecules in the chicken ceca between naïve uninfected control (N) and *Eimeria maxima* (EM) infected group.

## Data Availability

The datasets supporting the findings of this article are included within the article. The RNA-Seq raw data are available in the NCBI SRA repository under the Accession Number PRJNA540516.

## Abbreviations

EM: *Eimeria maxima*
N: naïve control
DEG: differentially expressed genes
KEGG: the Kyoto Encyclopedia of Genes and Genomes database
CD: cluster of differentiation
