# Supplementary material for: Eimeria maxima-induced transcriptional changes in the cecal mucosa of broiler chickens
Source: Parasit Vectors. 2019 Jun 4;12:285. doi: 10.1186/s13071-019-3534-4 (PMC6549307; doi:10.1186/s13071-019-3534-4)
Supplement: Supplementary file 1 — Additional file 1: Table S1. Data quality control summary. Table S2. The number of genes with different expression levels in Gallus gallus. Table S3. List of significantly upregulated genes in chicken cecal mucosa in response to Eimeria maxima infection. Table S4. List of significantly downregulated genes in chicken cecal mucosa in response to Eimeria maxima infection. Table S5. Gene expression changes for some pro-inflammatory molecules in the chicken ceca between naïve uninfected control (N) and Eimeria maxima (EM) infected group. [file 13071_2019_3534_MOESM1_ESM.docx]

**Additional file**

**Additional file 1: Table S1.** Data quality control summary

| **Sample Name** | **Group Name (biological replicates)** | **Raw reads** | **Clean Reads** | **Clean bases (data size)** | **Error rate (%)** | **Q20(%)** | **Q30(%)** | **GC Content(%)** |
| --- | --- | --- | --- | --- | --- | --- | --- | --- |
| rGC11 | Control | 125083126 | 120380388 | 18.06G | 0.02 | 96.51 | 91.62 | 48.32 |
| rGC12 |  | 121204928 | 116206764 | 17.43G | 0.02 | 96.5 | 91.34 | 49.91 |
| rGC13 |  | 123462742 | 118337220 | 17.75G | 0.02 | 96.44 | 91.2 | 48.95 |
| rGC31 | EM | 125140096 | 119896822 | 17.98G | 0.02 | 96.59 | 91.55 | 49.22 |
| rGC32 |  | 132748386 | 126306378 | 18.94G | 0.02 | 95.63 | 89.68 | 49.44 |
| rGC33 |  | 114987538 | 110026050 | 16.5G | 0.02 | 96.39 | 91.19 | 49.2 |

**Additional file 1: Table S2.** The number of genes with different expression levels in *Gallus gallus*

| **FPKM Interval** | **Naïve Control (N)** | | | ***Eimeria maxima* infected (EM)** | | |
| --- | --- | --- | --- | --- | --- | --- |
|  | **rGC11** | **rGC12** | **rGC13** | **rGC31** | **rGC32** | **rGC33** |
| 0~1 | 9729(39.39%) | 11180(45.26%) | 11428(46.27%) | 11468(46.43%) | 11398(46.15%) | 11597(46.95%) |
| 1~3 | 4144(16.78%) | 2999(12.14%) | 2570(10.40%) | 2771(11.22%) | 2588(10.48%) | 2682(10.86%) |
| 3~15 | 5861(23.73%) | 5822(23.57%) | 5550(22.47%) | 5389(21.82%) | 5536(22.41%) | 5412(21.91%) |
| 15~60 | 3815(15.45%) | 3635(14.72%) | 4021(16.28%) | 3900(15.79%) | 4016(16.26%) | 3882(15.72%) |
| >60 | 1151(4.66%) | 1064(4.31%) | 1131(4.58%) | 1172(4.74%) | 1162(4.70%) | 1127(4.56%) |

**Additional file 1: Table S3.** List of significantly upregulated genes in chicken cecal mucosa in response to *E. maxima* infection

| **Gene_ID** | **Chromosome** | **Strand** | **Fold changes** | **Adj p value** | **Description** |
| --- | --- | --- | --- | --- | --- |
| ABCA2 | 17 | - | 1.68 | 5.62E-03 | ABCA2_HUMAN ATP-binding cassette sub-family A member 2 |
| ABCA9 | 18 | - | 1.55 | 6.56E-03 | ABCA6_HUMAN ATP-binding cassette sub-family A member 6 |
| ABTB2 | 5 | - | 1.66 | 8.92E-03 | ABTB2_HUMAN Ankyrin repeat and BTB/POZ domain-containing protein 2 |
| ACP5 | 30 | - | 1.81 | 5.80E-04 | PPA5_MOUSE Tartrate-resistant acid phosphatase type 5 |
| ASAP3 | 23 | - | 1.61 | 9.00E-07 | ASAP3_HUMAN Arf-GAP with SH3 domain, ANK repeat and PH domain-containing protein 3 |
| ATG9B | 2 | + | 1.68 | 1.35E-02 | ATG9A_HUMAN Autophagy-related protein 9A |
| BF2 | 16 | - | 1.91 | 8.43E-05 | HA1F_CHICK Class I histocompatibility antigen, F10 alpha chain |
| BLB2 | 16 | + | 1.66 | 1.09E-02 | HB2L_CHICK Class II histocompatibility antigen, B-L beta chain (Fragment) |
| BTBD6 | 5 | + | 1.55 | 3.98E-02 | BTBD6_XENLA BTB/POZ domain-containing protein 6 |
| BTN3A3 | 31 | + | 1.80 | 1.24E-02 | BT2A1_HUMAN Butyrophilin subfamily 2 member A1 |
| C26H1orf106 | 26 | - | 1.68 | 6.13E-03 | INAVA_HUMAN Innate immunity activator protein |
| CAD | 3 | + | 1.62 | 3.19E-03 | PYR1_HUMAN CAD protein |
| CBX7 | 1 | + | 1.51 | 3.20E-03 | CBX7_HUMAN Chromobox protein homolog 7 |
| CCDC130 | 30 | - | 1.61 | 2.99E-02 | CC130_BOVIN Coiled-coil domain-containing protein 130 |
| CCL26 | 19 | + | 1.74 | 2.48E-03 | CCL3_PANTR C-C motif chemokine 3 |
| CDH3 | 11 | + | 1.55 | 1.81E-05 | CADHK_CHICK B-cadherin (Fragment) |
| CDKN1A | 26 | + | 1.64 | 1.73E-02 | CDN1A_MOUSE Cyclin-dependent kinase inhibitor 1 |
| CLCNKB | 21 | + | 1.64 | 3.87E-03 | CLCKB_XENLA Chloride channel protein ClC-Kb |
| CNGA4 | 1 | + | 1.72 | 4.59E-02 | CNGA4_MOUSE Cyclic nucleotide-gated cation channel alpha-4 |
| CPT1A | 5 | - | 1.81 | 2.72E-05 | CPT1A_HORSE Carnitine O-palmitoyltransferase 1, liver isoform |
| CTSS | 25 | - | 1.56 | 5.69E-05 | CATS_SAIBB Cathepsin S |
| CYP2C45 | 6 | + | 1.67 | 3.18E-03 | CP2C9_HUMAN Cytochrome P450 2C9 |
| CYR61 | 8 | - | 1.65 | 3.82E-02 | CYR61_CHICK Protein CYR61 |
| DALRD3 | 12 | + | 1.55 | 4.51E-03 | DALD3_MOUSE DALR anticodon-binding domain-containing protein 3 |
| DNASE2B | 8 | - | 1.62 | 1.47E-02 | DNS2B_HUMAN Deoxyribonuclease-2-beta |
| EGR1 | 13 | + | 1.67 | 2.57E-02 | EGR1_MOUSE Early growth response protein 1 |
| ERBB2 | 27 | + | 1.54 | 7.05E-04 | ERBB2_HUMAN Receptor tyrine-protein kinase erbB-2 |
| FAM155B | 4 | + | 1.51 | 1.63E-02 | F155B_XENTR Transmembrane protein FAM155B |
| FAM222B | 19 | - | 1.53 | 1.84E-02 | F222B_HUMAN Protein FAM222B |
| FES | 10 | - | 1.54 | 1.81E-02 | FPS_FUJSV Tyrine-protein kinase transforming protein Fps |
| FRMD8 | 33 | + | 1.68 | 1.09E-02 | FRMD8_XENTR FERM domain-containing protein 8 |
| GAAGSD | 14 | - | 1.51 | 1.76E-02 | LYAG_MOUSE Lysosomal alpha-glucosidase |
| GGCX | 22 | + | 1.52 | 2.38E-02 | VKGC_SHEEP Vitamin K-dependent gamma-carboxylase |
| GIMAP1 | 2 | + | 1.62 | 1.94E-03 | GIMA2_HUMAN GTPase IMAP family member 2 |
| GNLY | 22 | - | 1.60 | 3.95E-03 | NK-lysin |
| GPRC5A | 1 | + | 1.69 | 4.08E-03 | RAI3_HUMAN Retinoic acid-induced protein 3 |
| GPX4 | 28 | + | 1.60 | 6.73E-03 | GPX4_PIG Phospholipid hydroperoxide glutathione peroxidase |
| GRIN3B | 28 | + | 2.20 | 9.70E-06 | NMD3B_RAT Glutamate receptor ionotropic, NMDA 3B |
| GVINP1 | 3 | - | 1.81 | 5.07E-03 | GVIN1_HUMAN Interferon-induced very large GTPase 1 |
| GZMA | Z | + | 2.04 | 2.84E-04 | GRAA_BOVIN Granzyme A |
| HLA-F10AL4 | NW_020109758.1 | - | 5.32 | 5.26E-24 | HA1F_CHICK Class I histocompatibility antigen, F10 alpha chain |
| HTRA1 | 6 | + | 1.55 | 1.21E-02 | HTRA1_BOVIN Serine protease HTRA1 |
| IRF8 | 11 | - | 1.52 | 6.66E-03 | IRF8_CHICK Interferon regulatory factor 8 |
| ITGB6 | 7 | + | 1.62 | 3.95E-03 | ITB6_BOVIN Integrin beta-6 |
| JADE2 | 13 | - | 1.53 | 6.45E-03 | JADE2_MOUSE E3 ubiquitin-protein ligase Jade-2 |
| KBTBD11 | 3 | - | 1.65 | 2.37E-02 | KBTBB_MOUSE Kelch repeat and BTB domain-containing protein 11 |
| LIPA | 6 | - | 1.67 | 6.69E-04 | LICH_CROAD Putative lysosomal acid lipase/cholesteryl ester hydrolase |
| LOC100858737 | 26 | + | 1.62 | 1.93E-02 | SIM29_HUMAN Small integral membrane protein 29 |
| LOC100859837 | 1 | - | 1.79 | 2.07E-05 | SC6A7_MOUSE Sodium-dependent proline transporter |
| LOC101747455 | 18 | - | 3.71 | 2.09E-22 | POL_MMTVB Gag-Pro-Pol polyprotein |
| LOC107049129 | 5 | + | 1.75 | 9.84E-03 | C163A_CANLF Scavenger receptor cysteine-rich type 1 protein M130 |
| LOC107049500 | 23 | + | 1.93 | 1.04E-02 | PEX19_CRIGR Peroxisomal biogenesis factor 19 |
| LOC107050443 | 30 | + | 1.50 | 5.43E-03 | CTL2_MOUSE Choline transporter-like protein 2 |
| LOC107053113 | 3 | - | 1.59 | 4.01E-02 |  |
| LOC107053944 | 8 | + | 1.89 | 6.94E-03 |  |
| LOC107054704 | NW_020109758.1 | - | 5.60 | 6.31E-26 | HA1F_CHICK Class I histocompatibility antigen, F10 alpha chain |
| LOC107055478 | 31 | + | 1.75 | 3.13E-02 | BT2A1_HUMAN Butyrophilin subfamily 2 member A1 |
| LOC112529954 | 16 | - | 4.46 | 7.00E-16 | HA1F_CHICK Class I histocompatibility antigen, F10 alpha chain |
| LOC112529978 | 17 | + | 1.72 | 8.24E-03 | TPRN_RAT Taperin |
| LOC112530970 | 30 | - | 1.77 | 2.80E-02 |  |
| LOC112530987 | 30 | + | 1.89 | 6.94E-03 | PAR10_HUMAN Poly [ADP-ribe] polymerase 10 |
| LOC417013 | 15 | + | 1.53 | 1.69E-02 | ACD11_CAEEL Acyl-CoA dehydrogenase family member 11 |
| LOC417056 | NW_020109758.1 | - | 1.61 | 3.20E-02 | HA1F_CHICK Class I histocompatibility antigen, F10 alpha chain |
| LOC425607 | NW_020110167.1 | + | 1.51 | 6.94E-03 | GT251_BOVIN Procollagen galactyltransferase 1 |
| LOC776275 | 5 | - | 1.56 | 4.03E-02 | T263B_XENLA Transmembrane protein 263-B |
| LRFN1 | 32 | - | 1.64 | 1.14E-02 | LRFN1_HUMAN Leucine-rich repeat and fibronectin type III domain-containing protein 1 |
| LY96 | 2 | + | 1.67 | 3.10E-02 | LY96_HUMAN Lymphocyte antigen 96 |
| MCOLN1 | 30 | - | 1.56 | 4.56E-03 | MCLN1_MOUSE Mucolipin-1 |
| MGAT5B | 18 | - | 2.45 | 1.38E-07 | MGT5B_HUMAN Alpha-1,6-mannylglycoprotein 6-beta-N-acetylglucaminyltransferase B |
| MHCIY | 16 | - | 2.27 | 6.86E-06 | HA1F_CHICK Class I histocompatibility antigen, F10 alpha chain |
| MKNK2 | 28 | + | 1.61 | 3.35E-03 | MKNK2_RAT MAP kinase-interacting serine/threonine-protein kinase 2 |
| MST1 | 12 | + | 1.76 | 4.51E-03 | HGFL_HUMAN Hepatocyte growth factor-like protein |
| MTMR11 | 25 | + | 1.57 | 6.69E-04 | MTMRB_HUMAN Myotubularin-related protein 11 |
| NDRG1 | 2 | - | 1.63 | 1.12E-05 | NDRG1_MACFA Protein NDRG1 |
| NEURL1B | 13 | - | 1.61 | 1.10E-03 | NEU1B_HUMAN E3 ubiquitin-protein ligase NEURL1B |
| NIPAL2 | 2 | - | 1.74 | 7.43E-04 | NPAL2_MOUSE NIPA-like protein 2 |
| NOB1 | 11 | - | 1.50 | 3.20E-02 | NOB1_MACFA RNA-binding protein NOB1 |
| Novel00272 | 11 | + | 1.77 | 1.83E-03 |  |
| Novel00358 | 23 | + | 1.69 | 4.81E-02 |  |
| Novel00363 | 23 | - | 1.57 | 6.45E-03 |  |
| NPAS2 | 1 | + | 1.81 | 3.41E-05 | NPAS2_CHICK Neuronal PAS domain-containing protein 2 |
| OLFML2A | 17 | + | 1.59 | 3.42E-03 | OLM2A_XENTR Olfactomedin-like protein 2A |
| PDCD1LG2 | Z | + | 1.66 | 1.21E-02 | PD1L1_MOUSE Programmed cell death 1 ligand 1 |
| PDKI2 | 27 | - | 1.61 | 2.15E-02 | PDK2_MOUSE [Pyruvate dehydrogenase (acetyl-transferring)] kinase isozyme 2, mitochondrial |
| PPP1R1B | 27 | + | 1.69 | 1.40E-03 | PPR1B_BOVIN Protein phosphatase 1 regulatory subunit 1B |
| PROM1L | 6 | + | 1.52 | 1.27E-04 | PRM1A_DANRE Prominin-1-A |
| RALGDS | 17 | - | 1.50 | 1.43E-02 | GNDS_MOUSE Ral guanine nucleotide dissociation stimulator |
| RAPGEFL1 | 27 | + | 1.52 | 1.18E-02 | RPGFL_HUMAN Rap guanine nucleotide exchange factor-like 1 |
| RHOB | 3 | + | 1.78 | 1.77E-03 | RHOB_RAT Rho-related GTP-binding protein RhoB |
| RHPN1 | 2 | + | 1.55 | 1.09E-02 | RHPN1_HUMAN Rhophilin-1 |
| rna63493 | NC_001323.1 | - | 1.85 | 8.51E-05 | COX1_GADMO Cytochrome c oxidase subunit 1 |
| RPL22L1 | 9 | + | 1.51 | 1.76E-02 | RL22L_MOUSE 60S ribosomal protein L22-like 1 |
| RSFR | 6 | - | 1.60 | 2.59E-03 | RSFR_CHICK Ribonuclease homolog |
| RYBP | 12 | - | 1.58 | 4.07E-02 | RYBP_HUMAN RING1 and YY1-binding protein |
| SAMHD1 | 20 | - | 1.78 | 7.80E-06 | SAMH1_CHICK Deoxynucleoside triphosphate triphosphohydrolase SAMHD1 |
| SEMA3BL | 12 | + | 1.90 | 5.60E-07 | SEM3B_HUMAN Semaphorin-3B |
| SERPING1 | 5 | - | 1.57 | 8.36E-03 | IC1_HUMAN Plasma protease C1 inhibitor |
| SHROOM1 | 13 | - | 1.58 | 2.36E-02 | SHRM1_XENLA Protein Shroom1 |
| SHROOM2 | 1 | - | 1.50 | 3.15E-02 | SHRM2_XENTR Protein Shroom2 |
| SLC22A5 | 13 | + | 1.77 | 3.24E-11 | S22A5_HUMAN Solute carrier family 22 member 5 |
| SLC25A42 | 28 | - | 1.62 | 8.58E-03 | S2542_XENTR Mitochondrial coenzyme A transporter SLC25A42 |
| SLC26A4 | 1 | + | 1.57 | 1.73E-02 | S26A4_HUMAN Pendrin |
| SLC39A14 | 22 | - | 1.51 | 9.36E-03 | S39AE_HUMAN Zinc transporter ZIP14 |
| SMIM24 | 28 | + | 1.73 | 4.28E-02 |  |
| SOCS3 | 18 | + | 1.88 | 1.40E-03 | SOCS3_CHICK Suppressor of cytokine signaling 3 |
| SOX9 | 18 | + | 1.53 | 4.76E-02 | SOX9_CHICK Transcription factor SOX-9 |
| SP1 | 33 | + | 1.59 | 5.93E-04 | SP1_HUMAN Transcription factor Sp1 |
| SPHK1 | 18 | - | 1.50 | 7.63E-03 | SPHK1_MOUSE Sphingosine kinase 1 |
| SRCIN1 | 27 | - | 1.65 | 4.14E-03 | SRCN1_RAT SRC kinase signaling inhibitor 1 |
| TAP1 | 16 | - | 1.59 | 6.69E-04 | TAP1_GORGO Antigen peptide transporter 1 |
| TAPBP | 16 | - | 1.56 | 2.36E-04 | TPSN_CHICK Tapasin |
| TC2N | 5 | - | 1.57 | 6.93E-03 | TAC2N_HUMAN Tandem C2 domains nuclear protein |
| TCEA3 | 23 | - | 1.64 | 2.49E-03 | TCEA3_HUMAN Transcription elongation factor A protein 3 |
| TMC5 | 14 | + | 1.56 | 3.20E-03 | TMC5_RAT Transmembrane channel-like protein 5 |
| TMEM140 | 1 | - | 1.63 | 6.73E-03 | TM140_HUMAN Transmembrane protein 140 |
| TMEM82 | 21 | + | 1.51 | 7.31E-03 | TMM82_XENLA Transmembrane protein 82 |
| TNFRSF10B | 22 | + | 1.61 | 1.45E-02 | TR10B_HUMAN Tumor necrosis factor receptor superfamily member 10B |
| TNFSF10 | 9 | + | 1.50 | 6.66E-03 | TNF10_HUMAN Tumor necrosis factor ligand superfamily member 10 |
| TPP1 | 1 | - | 1.57 | 8.40E-05 | TPP1_MACFA Tripeptidyl-peptidase 1 |
| TRAF4 | 19 | + | 1.69 | 2.62E-03 | TRAF4_MOUSE TNF receptor-associated factor 4 |
| TRAIL-LIKE | 4 | - | 1.65 | 7.34E-04 | TNF10_MOUSE Tumor necrosis factor ligand superfamily member 10 |
| TRNP1 | 23 | + | 1.83 | 3.81E-03 |  |
| VNN1 | 3 | + | 1.59 | 3.29E-03 | VNN1_BOVIN Pantetheinase |
| VSIG4 | 4 | - | 1.71 | 4.91E-03 | VSIG4_HUMAN V-set and immunoglobulin domain-containing protein 4 |

* The ratio of Fragments Per Kilobase of transcript per Million mapped reads (fpkm) in EM group to that in N group (Fold changes ≥ 1.5, adjusted *p* ≤ 0.05).

**Additional file 1: Table S4.** List of significantly downregulated genes in chicken cecal mucosa in response to *E. maxima* infection

| **Gene_ID** | **Chromosome** | **Strand** | **Fold changes** | **Adj p value** | **Description** |
| --- | --- | --- | --- | --- | --- |
| ACAN | 10 | - | -2.03 | 0.0045148 | PGCA_CHICK Aggrecan core protein |
| ACE2 | 1 | + | -2.60 | 2.08E-05 | ACE2_FELCA Angiotensin-converting enzyme 2 |
| ACTG2 | 22 | + | -1.55 | 0.027218 | ACTH_RAT Actin, gamma-enteric smooth muscle |
| ADAMTS6 | Z | - | -1.58 | 0.012073 | ATS6_HUMAN A disintegrin and metalloproteinase with thrombospondin motifs 6 |
| ADGRB3 | 3 | - | -1.65 | 0.043446 | AGRB3_HUMAN Adhesion G protein-coupled receptor B3 |
| AFF3 | 1 | - | -1.71 | 0.0025321 | AFF3_HUMAN AF4/FMR2 family member 3 |
| AGBL1 | 10 | - | -1.92 | 0.010906 | CBPC4_HUMAN Cytosolic carboxypeptidase 4 |
| AMY2A | 8 | - | -1.87 | 0.014749 | AMYP_STRCA Pancreatic alpha-amylase |
| ANLN | 2 | - | -2.00 | 4.04E-07 | ANLN_HUMAN Anillin |
| AOX1 | 7 | + | -1.69 | 0.015721 | AOXA_HUMAN Aldehyde oxidase |
| AOX2 | 7 | + | -2.00 | 0.0017691 | AOXB_MOUSE Aldehyde oxidase 2 |
| APOB | 3 | - | -3.10 | 1.63E-08 | APOB_HUMAN Apolipoprotein B-100 |
| ATP2B1 | 1 | - | -1.61 | 6.67E-07 | AT2B1_MOUSE Plasma membrane calcium-transporting ATPase 1 |
| BBOX1 | 5 | + | -1.77 | 0.031319 | BODG_MOUSE Gamma-butyrobetaine dioxygenase |
| BIRC5 | 3 | - | -1.74 | 0.0079491 | BIRC5_BOVIN Baculoviral IAP repeat-containing protein 5 |
| BORL4 | 19 | - | -1.58 | 0.01284 | BORE1_CHICK Borealin |
| BPNT1 | 3 | + | -1.53 | 0.0007338 | BPNT1_BOVIN 3&ap;(2&ap;),5&ap;-bisphosssssssssssphate nucleotidase 1 |
| BT2A1L | 31 | + | -1.72 | 0.030986 | BT2A1_HUMAN Butyrophilin subfamily 2 member A1 |
| BUB1B | 5 | + | -1.65 | 0.011894 | BUB1B_HUMAN Mitotic checkpoint serine/threonine-protein kinase BUB1 beta |
| C9ORF152 | 2 | + | -1.71 | 0.049206 | CI152_HUMAN Uncharacterized protein C9orf152 |
| CACNA1C | 1 | + | -1.58 | 0.0085722 | CAC1C_HUMAN Voltage-dependent L-type calcium channel subunit alpha-1C |
| CACNA1I | 1 | - | -1.78 | 0.023743 | CAC1I_HUMAN Voltage-dependent T-type calcium channel subunit alpha-1I |
| CACNA2D4 | 1 | - | -1.77 | 0.016855 | CA2D4_HUMAN Voltage-dependent calcium channel subunit alpha-2/delta-4 |
| CALB1 | 2 | - | -3.25 | 1.92E-09 | CALB1_CHICK Calbindin |
| CAPN14 | 3 | + | -1.86 | 0.010775 | CAN14_HUMAN Calpain-14 |
| CAV1 | 1 | - | -1.58 | 0.035787 | CAV1_CHICK Caveolin-1 |
| CCDC170 | 3 | + | -1.72 | 0.045467 | CC170_HUMAN Coiled-coil domain-containing protein 170 |
| CCDC173 | 7 | + | -1.56 | 0.049561 | CC173_HUMAN Coiled-coil domain-containing protein 173 |
| CCNB2 | 10 | - | -1.74 | 0.0010961 | CCNB2_CHICK G2/mitotic-specific cyclin-B2 |
| CDC45 | 15 | + | -1.74 | 0.003947 | CDC45_HUMAN Cell division control protein 45 homolog |
| CDK1 | 6 | - | -1.54 | 0.038747 | CDK1_CHICK Cyclin-dependent kinase 1 |
| CELSR1 | 1 | - | -1.72 | 0.039621 | CELR1_MOUSE Cadherin EGF LAG seven-pass G-type receptor 1 |
| CENPE | 4 | - | -1.93 | 2.18E-05 | CENPE_HUMAN Centromere-associated protein E |
| CENPF | 3 | - | -1.88 | 2.35E-05 | CENPF_HUMAN Centromere protein F |
| CENPK | Z | - | -1.61 | 0.043446 | CENPK_CHICK Centromere protein K |
| CENPW | 3 | - | -1.94 | 0.0057137 | CENPW_CHICK Centromere protein W |
| CKAP2 | 1 | - | -1.54 | 0.014334 | CKAP2_HUMAN Cytoskeleton-associated protein 2 |
| COL1A1 | 27 | + | -1.66 | 0.014494 | CO1A1_CHICK Collagen alpha-1(I) chain |
| COL3A1 | 7 | - | -1.50 | 0.039146 | CO3A1_CHICK Collagen alpha-1(III) chain (Fragments) |
| COL8A2 | 23 | + | -1.51 | 0.042671 | CO8A2_HUMAN Collagen alpha-2(VIII) chain |
| COX7A2 | 3 | + | -1.52 | 3.63E-05 | CX7A2_RAT Cytochrome c oxidase subunit 7A2, mitochondrial |
| CRTAC1 | 6 | + | -1.75 | 0.034856 | CRAC1_HUMAN Cartilage acidic protein 1 |
| CSMD1 | 3 | + | -1.82 | 0.018027 | CSMD1_HUMAN CUB and sushi domain-containing protein 1 |
| CUBN | 2 | + | -1.85 | 0.016953 | CUBN_CANLF Cubilin |
| CYCS | 2 | - | -1.50 | 0.004106 | CYC_MELGA Cytochrome c |
| CYP2C23a | 6 | - | -2.80 | 7.09E-10 | CP2H1_CHICK Cytochrome P450 2H1 |
| CYP2C23b | 6 | - | -2.81 | 7.42E-07 | CP2H2_CHICK Cytochrome P450 2H2 |
| DIAPH3 | 1 | + | -1.63 | 0.022514 | DIAP3_HUMAN Protein diaphanous homolog 3 |
| DLG2 | 1 | - | -1.76 | 0.0125 | DLG2_HUMAN Disks large homolog 2 |
| DNAH1 | 12 | + | -1.74 | 0.02114 | DYH1_HUMAN Dynein heavy chain 1, axonemal |
| DNAH7 | 7 | - | -1.70 | 0.039278 | DYH7_HUMAN Dynein heavy chain 7, axonemal |
| DNAH8 | 3 | - | -1.69 | 0.039146 | DYH8_MOUSE Dynein heavy chain 8, axonemal |
| DNAJA3 | 14 | + | -1.55 | 0.014349 | DNJA3_HUMAN DnaJ homolog subfamily A member 3, mitochondrial |
| DPP10 | 7 | - | -1.81 | 0.021608 | DPP10_HUMAN Inactive dipeptidyl peptidase 10 |
| EPHA7 | 3 | + | -1.72 | 0.037002 | EPHA7_CHICK Ephrin type-A receptor 7 |
| ETNPPL | 4 | + | -1.79 | 0.027407 | AT2L1_XENLA Ethanolamine-phosphate phosphor-lyase |
| FABP2 | 4 | + | -3.73 | 7.00E-16 | FABPI_MOUSE Fatty acid-binding protein, intestinal |
| FANCD2 | 12 | - | -1.68 | 0.014488 | FACD2_HUMAN Fanconi anemia group D2 protein |
| FBN2 | Z | - | -2.16 | 0.0013384 | FBN2_HUMAN Fibrillin-2 |
| FER1L4 | 20 | + | -2.18 | 0.0012542 | FR1L4_MOUSE Fer-1-like protein 4 |
| FHL5 | 3 | - | -1.50 | 0.0125 | FHL5_BOVIN Four and a half LIM domains protein 5 |
| FIBIN | 5 | + | -1.67 | 0.029086 | FIBIN_BOVIN Fin bud initiation factor homolog |
| FREM1 | Z | - | -1.59 | 0.023626 | FREM1_HUMAN FRAS1-related extracellular matrix protein 1 |
| FTCD | 7 | - | -1.68 | 6.15E-06 | FTCD_CHICK Formimidoyltransferase-cyclodeaminase |
| GABRP | 13 | - | -1.76 | 0.02754 | GBRP_HUMAN Gamma-aminobutyric acid receptor subunit pi |
| GABRR1 | 3 | + | -1.71 | 0.021474 | GBRR1_RAT Gamma-aminobutyric acid receptor subunit rho-1 |
| GJA8 | 1 | + | -1.96 | 0.0069439 | CXA8_CHICK Gap junction alpha-8 protein |
| GNAO1 | 11 | + | -1.64 | 0.038998 | GNAO_XENLA Guanine nucleotide-binding protein G(o) subunit alpha |
| GPC6 | 1 | - | -1.67 | 0.042671 | GPC6_HUMAN Glypican-6 |
| GRHL3 | 23 | + | -2.75 | 1.37E-07 | GRHL3_MOUSE Grainyhead-like protein 3 homolog |
| GRIA4 | 1 | - | -1.77 | 0.019007 | GRIA4_RAT Glutamate receptor 4 |
| GRIN2C | 18 | - | -1.68 | 0.043446 | NMDE3_MOUSE Glutamate receptor ionotropic, NMDA 2C |
| GSTA2 | 3 | + | -1.68 | 0.0043431 | GSTA2_CHICK Glutathione S-transferase |
| GSTA3 | 3 | + | -1.69 | 0.0039224 | GSTA1_CHICK Glutathione S-transferase |
| GSTO1 | 6 | + | -1.93 | 0.0006757 | GSTO1_HUMAN Glutathione S-transferase omega-1 |
| HDAC9 | 2 | + | -1.54 | 0.043552 | HDAC9_HUMAN Histone deacetylase 9 |
| HGFAC | 4 | - | -1.64 | 0.024157 | HGFA_MOUSE Hepatocyte growth factor activator |
| HIST1H110 | 1 | - | -1.51 | 0.0085917 | H110_CHICK Histone H1.10 |
| HIST1H2A4L3 | 1 | - | -2.83 | 1.37E-07 | H2A_CAIMO Histone H2A |
| HIST1H2B8 | 1 | + | -1.60 | 0.011988 | H2B2E_PONAB Histone H2B type 2-E |
| HISTH2A4L1 | 1 | - | -1.66 | 0.0003416 | H2AJ_HUMAN Histone H2A.J |
| HMMR | 13 | - | -1.71 | 0.0079491 | HMMR_HUMAN Hyaluronan mediated motility receptor |
| HS6ST3 | 1 | - | -1.87 | 0.016783 | H6ST3_MOUSE Heparan-sulfate 6-O-sulfotransferase 3 |
| HSPA2 | 5 | - | -1.50 | 0.0021141 | HSP70_CHICK Heat shock 70 kDa protein |
| HSPA5 | 17 | - | -1.60 | 0.0023992 | BIP_CHICK Endoplasmic reticulum chaperone BiP |
| KCNE1 | 1 | - | -2.96 | 7.97E-15 | KCNE1_HUMAN Potassium voltage-gated channel subfamily E member 1 |
| KIAA1024 | 10 | - | -1.73 | 0.034663 | MNAR1_HUMAN Major intrinsically disordered Notch2-binding receptor 1 |
| KIF11 | 6 | - | -1.55 | 0.007667 | KIF11_XENTR Kinesin-like protein KIF11 |
| KIF14 | 8 | + | -1.66 | 0.0094865 | KIF14_HUMAN Kinesin-like protein KIF14 |
| KLF9 | Z | - | -1.96 | 1.65E-05 | KLF9_RAT Krueppel-like factor 9 |
| KPNA2 | 18 | - | -1.85 | 0.0001952 | IMA1_HUMAN Importin subunit alpha-1 |
| LAMB4 | 1 | - | -1.70 | 0.049622 | LAMB4_HUMAN Laminin subunit beta-4 |
| LBH | 3 | - | -1.60 | 0.0004295 | LBH_CHICK Protein LBH |
| LINGO2 | Z | - | -1.61 | 0.013007 | LIGO2_HUMAN Leucine-rich repeat and immunoglobulin-like domain-containing nogo receptor-interacting protein 2 |
| LOC100859246 | 3 | - | -1.80 | 0.020201 |  |
| LOC100859492 | 2 | + | -1.75 | 0.032962 |  |
| LOC101747340 | 9 | + | -1.87 | 0.0034167 |  |
| LOC101747448 | 9 | - | -1.89 | 0.014021 |  |
| LOC101747954 | 6 | - | -1.78 | 0.031964 |  |
| LOC101748427 | 12 | + | -1.69 | 0.033053 |  |
| LOC101748713 | 1 | - | -1.69 | 0.039278 |  |
| LOC101748799 | 7 | + | -1.79 | 0.021608 |  |
| LOC101748814 | 4 | + | -1.79 | 0.023626 |  |
| LOC101749216 | 13 | - | -1.74 | 0.039049 | ISK1_STRCA Pancreatic secretory trypsin inhibitor |
| LOC101749453 | 5 | - | -1.60 | 0.040356 | FBX16_HUMAN F-box only protein 16 |
| LOC101749477 | 9 | + | -1.84 | 0.020579 |  |
| LOC101749621 | 2 | + | -1.72 | 0.029127 |  |
| LOC101749691 | 2 | - | -1.68 | 0.032387 |  |
| LOC101749793 | 4 | + | -1.73 | 0.042476 |  |
| LOC101750098 | 6 | - | -1.84 | 0.016783 |  |
| LOC101750125 | 3 | + | -1.66 | 0.042671 |  |
| LOC101750386 | 14 | - | -1.72 | 0.040211 |  |
| LOC101750826 | 1 | + | -1.69 | 0.023626 |  |
| LOC101751305 | 31 | - | -1.69 | 0.025821 | sarcolemmal membrane-associated protein-like |
| LOC101751795 | 20 | + | -1.93 | 0.0090703 |  |
| LOC101752258 | 3 | - | -1.72 | 0.028768 |  |
| LOC107049364 | 1 | - | -1.83 | 0.021733 | RHG32_MOUSE Rho GTPase-activating protein 32 |
| LOC107049551 | 1 | - | -1.87 | 0.012071 | MRO2B_MOUSE Maestro heat-like repeat-containing protein family member 2B |
| LOC107051247 | 1 | + | -1.64 | 0.042366 |  |
| LOC107051649 | 2 | - | -1.61 | 0.048052 |  |
| LOC107051651 | 2 | - | -1.79 | 0.020417 |  |
| LOC107051926 | Z | + | -1.81 | 0.015817 |  |
| LOC107052089 | 4 | - | -2.06 | 0.0021551 |  |
| LOC107052435 | Z | + | -1.70 | 0.037297 |  |
| LOC107053395 | 5 | - | -1.74 | 0.024851 |  |
| LOC107053481 | 1 | + | -1.73 | 0.012073 |  |
| LOC107053554 | 5 | + | -1.69 | 0.034609 |  |
| LOC107053572 | 5 | + | -1.75 | 0.030559 |  |
| LOC107053701 | 1 | + | -1.86 | 0.01506 | G2E3_MOUSE G2/M phase-specific E3 ubiquitin-protein ligase |
| LOC107053789 | 7 | + | -1.70 | 0.043724 |  |
| LOC107053801 | 7 | + | -1.83 | 0.021474 |  |
| LOC107053888 | 7 | - | -1.94 | 0.0076279 |  |
| LOC107053912 | 8 | + | -2.26 | 4.21E-06 |  |
| LOC107053913 | 8 | + | -1.81 | 1.72E-05 |  |
| LOC107053936 | 8 | - | -1.79 | 0.011417 |  |
| LOC107053990 | 8 | + | -1.75 | 0.02754 |  |
| LOC107054047 | 8 | + | -1.72 | 0.044776 |  |
| LOC107054130 | 9 | - | -2.04 | 0.003947 |  |
| LOC107054137 | 9 | - | -1.69 | 0.042286 |  |
| LOC107054158 | 10 | + | -1.81 | 0.025884 | KRF1_COLLI Feather keratin C1-1/C1-3/C2-1 |
| LOC107054170 | 1 | + | -1.95 | 0.0066814 |  |
| LOC107054324 | 11 | - | -1.72 | 0.023626 |  |
| LOC107054551 | 13 | - | -1.64 | 0.044776 |  |
| LOC107054639 | 14 | + | -1.69 | 0.041647 |  |
| LOC107054644 | 1 | + | -1.84 | 0.010215 |  |
| LOC107054831 | 20 | + | -1.69 | 0.040802 |  |
| LOC107055306 | 1 | + | -1.63 | 0.023626 |  |
| LOC107055587 | 1 | + | -1.74 | 0.041136 | RBP2_MOUSE E3 SUMO-protein ligase RanBP2 |
| LOC107055643 | 1 | + | -1.72 | 0.014533 | MRO2B_MOUSE Maestro heat-like repeat-containing protein family member 2B |
| LOC107056422 | 5 | - | -1.72 | 0.019318 | IPIL1_RAT Initol 1,4,5-trisphphate receptor-interacting protein-like 1 |
| LOC107056869 | Z | - | -1.75 | 0.019547 | G2/M phase-specific E3 ubiquitin-protein ligase-like |
| LOC107056924 | 1 | + | -1.89 | 0.0098382 | MRO2B_MOUSE Maestro heat-like repeat-containing protein family member 2B |
| LOC107057081 | 1 | - | -1.76 | 0.031546 |  |
| LOC107057257 | NW_020110167.1 | + | -1.82 | 0.023626 | ER1_BOVIN Oxidative stress-responsive serine-rich protein 1 |
| LOC112530112 | 20 | - | -1.78 | 0.025794 |  |
| LOC112530221 | 23 | + | -1.79 | 0.0082674 |  |
| LOC112530274 | 24 | - | -1.73 | 0.040876 |  |
| LOC112530392 | 27 | + | -1.85 | 0.015309 |  |
| LOC112530523 | 1 | + | -1.96 | 0.0019367 |  |
| LOC112530580 | Z | - | -1.74 | 0.038747 |  |
| LOC112530621 | Z | - | -2.36 | 0.0002133 | ENV_AVISU Envelope glycoprotein (Fragment) |
| LOC112531432 | NW_020110165.1 | - | -1.79 | 0.023626 |  |
| LOC112531480 | NW_020110167.1 | - | -1.80 | 0.025255 |  |
| LOC112531499 | 1 | + | -1.71 | 0.042174 | M1T_DROMA Mariner M1 transpase |
| LOC112531773 | 2 | - | -1.56 | 0.025013 |  |
| LOC112531817 | 2 | - | -1.90 | 0.012352 |  |
| LOC112531860 | 2 | + | -1.88 | 0.01517 |  |
| LOC112531897 | 2 | + | -1.50 | 0.040653 |  |
| LOC112531906 | 2 | - | -2.43 | 8.50E-05 | GAG_RSVP Gag polyprotein |
| LOC112532104 | 3 | + | -1.72 | 0.024613 |  |
| LOC112532320 | 4 | - | -1.86 | 0.0067991 |  |
| LOC112532346 | 4 | - | -1.74 | 0.01941 |  |
| LOC112532365 | 4 | - | -1.77 | 0.030859 |  |
| LOC112532395 | 4 | + | -1.80 | 0.012516 |  |
| LOC112532397 | 4 | + | -1.74 | 0.013933 |  |
| LOC112532422 | 4 | + | -1.74 | 0.041846 |  |
| LOC112532700 | 6 | + | -2.23 | 0.0008104 | DMBT1_HUMAN Deleted in malignant brain tumors 1 protein |
| LOC112532749 | 1 | - | -1.59 | 0.040653 |  |
| LOC112532931 | 1 | - | -1.64 | 0.043724 |  |
| LOC112533025 | 9 | + | -2.03 | 0.0034185 |  |
| LOC112533490 | 1 | - | -1.65 | 0.046956 |  |
| LOC112533599 | 16 | + | -1.81 | 0.015901 | RRT15_YEAST Regulator of rDNA transcription protein 15 |
| LOC112533601 | 16 | + | -3.98 | 3.98E-12 | NLRC3_MOUSE Protein NLRC3 |
| LOC112533602 | 16 | + | -2.99 | 2.77E-07 |  |
| LOC112533603 | 16 | + | -1.65 | 0.025656 | NLRC3_MOUSE Protein NLRC3 |
| LOC420807 | 2 | - | -1.66 | 0.020417 | TKRA_BACSU Probable 2-ketogluconate reductase |
| LOC768995 | 4 | + | -1.71 | 0.043446 |  |
| LOR1 | 25 | + | -1.62 | 0.049604 |  |
| LOXL4 | 6 | + | -1.78 | 0.030986 | LOXL4_BOVIN Lysyl oxidase homolog 4 |
| LRRC17 | 1 | + | -1.64 | 0.048271 | LRC17_HUMAN Leucine-rich repeat-containing protein 17 |
| MAP1B | Z | - | -1.85 | 0.010906 | MAP1B_RAT Microtubule-associated protein 1B |
| MCT2L | 12 | + | -2.76 | 3.18E-06 | MOT2_HUMAN Monocarboxylate transporter 2 |
| ME3 | 1 | + | -1.69 | 0.045467 | MAON_MOUSE NADP-dependent malic enzyme, mitochondrial |
| MFAP5 | 1 | - | -1.82 | 0.0065296 | MFAP5_MOUSE Microfibrillar-associated protein 5 |
| MGAM | NW_020110167.1 | - | -1.83 | 0.018222 | MGA_HUMAN Maltase-glucoamylase, intestinal |
| MHCIA9 | 16 | + | -1.53 | 0.03975 | HA1F_CHICK Class I histocompatibility antigen, F10 alpha chain |
| MKI67 | 6 | + | -1.56 | 0.0002191 | KI67_HUMAN Proliferation marker protein Ki-67 |
| MRM2 | 14 | - | -2.01 | 0.0020348 | MRM2_HUMAN rRNA methyltransferase 2, mitochondrial |
| MSMB | 6 | + | -1.73 | 0.043382 | MSPA_SAGOE Beta-micreminoprotein A1 |
| MTFR2 | 3 | + | -1.66 | 0.038747 | MTFR2_HUMAN Mitochondrial fission regulator 2 |
| MTTP | 4 | + | -1.86 | 0.014838 | MTP_MESAU Microsomal triglyceride transfer protein large subunit |
| MYL1 | 7 | - | -1.95 | 0.0084917 | MLE1_CHICK Myin light chain 1, skeletal muscle isoform |
| MYO3B | 7 | - | -1.79 | 0.027249 | MYO3B_HUMAN Myin-IIIb |
| MYO7A | 1 | - | -1.70 | 0.045059 | MYO7A_HUMAN Unconventional myin-VIIa |
| NCAPG2 | 2 | - | -1.71 | 0.0075317 | CNDG2_XENLA Condensin-2 complex subunit G2 |
| NDC80 | 2 | - | -1.66 | 0.022362 | NDC80_CHICK Kinetochore protein NDC80 homolog |
| NELL2 | 1 | - | -2.21 | 0.0005925 | NEL_CHICK Protein NEL |
| NMRK2 | 28 | - | -1.55 | 0.041674 | NRK2_HUMAN Nicotinamide ribide kinase 2 |
| NOP2 | 1 | - | -1.76 | 0.0049553 | NOP2_HUMAN Probable 28S rRNA (cytine(4447)-C(5))-methyltransferase |
| Novel00040 | 1 | - | -1.72 | 0.044776 |  |
| Novel00049 | 1 | - | -2.15 | 0.0014506 |  |
| Novel00082 | 2 | + | -1.75 | 0.037238 | IPIL1_MOUSE Initol 1,4,5-trisphphate receptor-interacting protein-like 1 |
| Novel00104 | 2 | - | -1.86 | 0.015525 | IPIL1_RAT Initol 1,4,5-trisphphate receptor-interacting protein-like 1 |
| Novel00129 | 3 | - | -1.73 | 0.021608 | PLB1_MONDO Phospholipase B1, membrane-associated |
| Novel00167 | 4 | - | -1.54 | 0.017684 |  |
| Novel00202 | 6 | - | -2.11 | 0.0022165 |  |
| Novel00225 | 8 | + | -2.26 | 0.0004792 |  |
| Novel00231 | 8 | + | -1.86 | 0.0084116 |  |
| Novel00243 | 8 | - | -1.93 | 0.0098844 |  |
| Novel00250 | 9 | + | -2.23 | 0.0008037 |  |
| Novel00269 | 10 | - | -1.63 | 0.03986 |  |
| Novel00271 | 11 | + | -1.86 | 0.01506 |  |
| Novel00282 | 12 | + | -1.95 | 0.0088188 |  |
| Novel00296 | 14 | + | -1.88 | 0.0075317 |  |
| Novel00301 | 14 | - | -2.34 | 0.000191 | NES, paranemin; nestin; K07609 nestin |
| Novel00303 | 14 | - | -2.06 | 0.0038711 |  |
| Novel00305 | 14 | - | -1.71 | 0.046541 |  |
| Novel00324 | 18 | + | -1.65 | 0.047378 |  |
| Novel00326 | 18 | + | -1.60 | 0.043357 |  |
| Novel00370 | 25 | + | -2.14 | 0.0016463 |  |
| Novel00373 | 25 | - | -2.39 | 3.82E-05 |  |
| Novel00384 | 27 | + | -1.93 | 0.0093626 |  |
| Novel00388 | 27 | + | -1.69 | 0.030837 |  |
| Novel00389 | 27 | + | -1.73 | 0.028836 |  |
| Novel00395 | 27 | + | -2.85 | 9.00E-07 |  |
| Novel00466 | 33 | + | -1.77 | 0.027249 |  |
| Novel00470 | 33 | + | -2.20 | 0.0003679 |  |
| Novel00494 | 31 | - | -1.73 | 0.038421 |  |
| Novel00506 | NW_020109838.1 | + | -1.78 | 0.030476 |  |
| Novel00508 | NW_020109883.1 | + | -2.45 | 3.41E-05 |  |
| Novel00515 | NW_020110000.1 | + | -1.74 | 0.039858 |  |
| Novel00518 | NW_020110058.1 | - | -1.78 | 0.028836 |  |
| Novel00519 | NW_020110062.1 | + | -1.78 | 0.030859 |  |
| Novel00524 | NW_020110136.1 | + | -1.61 | 0.034609 |  |
| Novel00530 | NW_020110158.1 | + | -1.74 | 0.041647 |  |
| Novel00531 | NW_020110158.1 | - | -1.90 | 0.01284 |  |
| Novel00537 | NW_020110161.1 | + | -1.82 | 0.0089164 |  |
| Novel00539 | NW_020110161.1 | - | -1.80 | 0.017797 |  |
| Novel00540 | NW_020110161.1 | - | -2.26 | 0.0006403 |  |
| Novel00560 | NW_020110165.1 | + | -2.06 | 0.0038711 |  |
| NREP | Z | + | -1.55 | 0.014628 | NREP_CHICK Neuronal regeneration-related protein |
| NT5C1A | 23 | - | -1.74 | 0.032114 | 5NT1A_MOUSE Cytolic 5&ap;-nucleotidase 1A |
| NUSAP1 | 5 | + | -1.55 | 0.010938 | NUSAP_CHICK Nucleolar and spindle-associated protein 1 |
| NWD2 | 4 | - | -1.91 | 0.0030083 | NWD2_HUMAN NACHT and WD repeat domain-containing protein 2 |
| OAT | 6 | - | -1.50 | 0.021474 | OAT_BOVIN Ornithine aminotransferase, mitochondrial |
| OTOF | 3 | + | -1.96 | 0.0075317 | OTOF_RAT Otoferlin |
| PI15 | 2 | + | -2.43 | 3.63E-05 | PI15_CHICK Peptidase inhibitor 15 |
| PLA2G4EL2 | 5 | - | -1.83 | 0.017279 | PA24E_HUMAN Cytolic phospholipase A2 epsilon |
| PLK1 | 14 | + | -1.59 | 0.030476 | PLK1_MOUSE Serine/threonine-protein kinase PLK1 |
| PPL | 14 | - | -1.90 | 0.012851 | PEPL_HUMAN Periplakin |
| PRG4 | 8 | - | -1.75 | 0.038505 | PRG4_HUMAN Proteoglycan 4 |
| PRSS23 | 1 | - | -1.55 | 0.003975 | PRS23_BOVIN Serine protease 23 |
| PTPN5 | 5 | - | -1.78 | 0.027249 | PTN5_HUMAN Tyrine-protein phosphatase non-receptor type 5 |
| rna63480 | NC_001323.1 | + | -2.12 | 0.0021551 |  |
| rna63481 | NC_001323.1 | + | -3.72 | 6.48E-11 |  |
| rna63484 | NC_001323.1 | + | -1.82 | 0.015817 |  |
| rna63487 | NC_001323.1 | + | -1.72 | 0.034558 |  |
| rna63497 | NC_001323.1 | + | -1.72 | 0.03202 |  |
| rna63498 | NC_001323.1 | + | -1.86 | 0.016666 |  |
| rna63501 | NC_001323.1 | + | -1.71 | 0.042366 |  |
| RRM2 | 3 | + | -1.81 | 0.0001386 | RIR2_HUMAN Ribonucleoside-diphosphate reductase subunit M2 |
| RRP12 | 6 | + | -4.09 | 2.10E-17 | RRP12_CHICK RRP12-like protein |
| RRP15 | 3 | - | -2.82 | 1.26E-06 | RRP15_HUMAN RRP15-like protein |
| RRP1B | 1 | + | -8.04 | 4.86E-56 | RRP1B_HUMAN ribosomal RNA processing protein 1 homolog B |
| RRP36 | 3 | + | -6.49 | 4.57E-34 | RRP36_BOVIN ribosomal RNA processing protein 36 homolog |
| RRP7A | 1 | + | -4.57 | 2.05E-18 | RRP7A_MOUSE ribosomal RNA-processing protein 7 homolog A |
| RRP9 | 12 | + | -2.52 | 4.26E-07 | U3IP2_MOUSE U3 small nucleolar RNA-interacting protein 2 |
| RYR2 | 3 | - | -1.74 | 0.038747 | RYR2_HUMAN Ryanodine receptor 2 |
| SCD | 6 | - | -2.31 | 4.78E-05 | ACOD_CYPCA Acyl-CoA desaturase |
| SCUBE2 | 5 | + | -2.18 | 0.000186 | SCUB2_HUMAN Signal peptide, CUB and EGF-like domain-containing protein 2 |
| SGCG | 1 | - | -1.62 | 0.041165 | SGCG_CANLF Gamma-sarcoglycan |
| SHANK2 | 5 | - | -1.95 | 0.0083613 | SHAN2_HUMAN SH3 and multiple ankyrin repeat domains protein 2 |
| SIIL | 1 | + | -1.72 | 0.025656 | SUIS_RABIT Sucrase-isomaltase, intestinal |
| SLC15A1 | 1 | + | -1.79 | 0.022514 | S15A1_MOUSE Solute carrier family 15 member 1 |
| SLC22A3 | 3 | - | -1.65 | 0.033723 | S22A3_MOUSE Solute carrier family 22 member 3 |
| SLC25A47 | 5 | + | -1.72 | 0.048079 | S2547_MOUSE Solute carrier family 25 member 47 |
| SLC6A12 | 1 | - | -1.75 | 0.025013 | S6A12_HUMAN Sodium- and chloride-dependent betaine transporter |
| SLIT3 | 13 | + | -1.77 | 0.0024232 | SLIT3_RAT Slit homolog 3 protein |
| SMC2 | Z | - | -1.60 | 0.0006688 | SMC2_CHICK Structural maintenance of chromosomes protein 2 |
| SMIM5 | 18 | - | -2.08 | 9.40E-05 | SMIM5; small integral membrane protein 5 |
| SPC25 | 7 | + | -1.63 | 0.040141 | SPC25_BOVIN Kinetochore protein Spc25 |
| SSPO | 2 | + | -1.87 | 0.014576 | SSPO_CHICK SCO-spondin |
| STC2 | 13 | - | -1.84 | 0.0089164 | STC2_PONAB Stanniocalcin-2 |
| STMN1 | 23 | + | -1.54 | 0.0041666 | STMN1_CHICK Stathmin |
| SULT1C3 | 1 | + | -1.54 | 0.0006252 | ST1C1_RAT Sulfotransferase 1C1 |
| TENM2 | 13 | - | -2.12 | 0.0021141 | TEN2_CHICK Teneurin-2 |
| TFAP2D | 3 | - | -1.64 | 0.049114 | AP2D_HUMAN Transcription factor AP-2-delta |
| TPD52 | 2 | - | -1.55 | 0.0016564 | TPD52_RABIT Tumor protein D52 |
| TTK | 3 | - | -1.53 | 0.033053 | TTK_HUMAN Dual specificity protein kinase TTK |
| TUBA1A | 33 | + | -1.64 | 0.0010961 | TBA1A_RAT Tubulin alpha-1A chain |
| UPP1 | 2 | + | -1.87 | 0.007667 | UPP1_MOUSE Uridine phosphorylase 1 |
| USH2A | 3 | + | -1.81 | 0.024613 | USH2A_HUMAN Usherin |
| VSTM2A | 2 | + | -1.72 | 0.028514 | VTM2A_HUMAN V-set and transmembrane domain-containing protein 2A |
| WNK2 | 12 | - | -1.76 | 0.031964 | WNK2_MOUSE Serine/threonine-protein kinase WNK2 |
| ZONADHL | 24 | + | -1.79 | 0.014021 | SCN3B_BOVIN Sodium channel subunit beta-3 |

* The ratio of Fragments Per Kilobase of transcript per Million mapped reads (fpkm) in EM group to that in N group (|Fold changes| ≥ 1.5), adjust p value ≤ 0.05).

**Additional file 1: Table S5**. Gene expression changes for some pro-inflammatory molecules in the chicken ceca between Naïve uninfected control (N) and *Eimeria maxima* (EM) infected group

| **Gene_ID#** | **Fold change***  **(EM vs N)** | **Protein** | **Detailed description** |
| --- | --- | --- | --- |
| IL6 | 0.94 | IL6 | TB2/DP1/HVA22-related protein |
| TNFRSF10B | 1.61 | TNFRSF10B | Tumor necrosis factor Receptor Superfamily Member 10b) |
| VEGFC | 1.08 | VEGFC | Vascular endothelial growth factor C precursor |
| CD28 | 1.24 | CD28 | T cell antigen CD28 |
| LITAF | 1.21 | LITAF | LPS-induced tumor necrosis factor alpha factor |

*The ratio of Fragments Per Kilobase of transcript per Million mapped reads (fpkm) in EM group to that in N group. In RNA-Seq, the relative expression of a transcript is proportional to the number of cDNA fragments that originate from it.
